# A comparative study of the effect of capsular repair in the Latarjet procedure

**DOI:** 10.1186/s12891-025-08278-8

**Published:** 2025-01-25

**Authors:** Andrew P. McBride, Thibault Lafosse, Alex N. Karanja, Laurent Lafosse, Ian P. Hughes, Gregory A. Hoy, Eugene T. Ek, Shane A. Barwood, Fraser Taylor, Ezekiel Tan

**Affiliations:** 1 Gold Coast University Hospital, 1 Hospital Boulevard, Gold Coast, Southport, QLD 4215 Australia; 2 Melbourne Orthopaedic Group, Windsor, Melbourne, VIC Australia; 3 Alps Surgical Institute, Annecy, France; 4 School of Medicine, University of Queensland, Brisbane, QLD Australia

**Keywords:** Shoulder instability, Latarjet procedure, Shoulder arthroscopy, Range of motion, Proprioception, Outcome

## Abstract

**Background:**

Long term studies have shown the Latarjet procedure to be successful in preventing re-dislocation in primary and recurrent anterior inferior shoulder instability. It provides stability through the sling effect of the conjoint tendon and the bone block. It is unclear whether augmentation with capsular repair provides an added benefit or leads to restricted range of external rotation. The primary aim of this study is to evaluate the effect of capsular repair in the open Latarjet procedure on rotational range of active external rotation in 90 degrees abduction (RoM-ER90). The secondary aim is to evaluate the effect on clinical outcomes including post-operative apprehension, instability, proprioception and shoulder function scores.

**Methods:**

This is a multi-national retrospective cohort study including patients with a minimum of 6-months follow-up post Latarjet procedure performed between 2016 and 2020 recruited from 3 units in Australia and France. Range of motion was measured using a Proteck goniometer. Clinical outcomes were assessed using the Western Ontario Shoulder Instability Index (WOSI), Oxford shoulder, Oxford instability, Walch-Duplay and Rowe scores. Shoulder proprioception was assessed by the active relocation test described by Glendon et al.

**Results:**

Forty-four patients were included, median age was 29.5 years and 91% male. Three groups were assessed, open latarjet with no capsular repair (OL *n* = 11), open latarjet with capsular repair (OLCR *n* = 20), and arthroscopic Latarjet without capsular repair (AL *n* = 13). There was no apparent effect of capsular repair on the ROM-ER 90 in the open groups with a median (interquartile range) of 78° (72°, 90°) for OL and 84° (75°, 90°; *P* = 0.87) for OLCR groups. Capsular repair and arthroscopic approach did not affect the proportion of patients reporting shoulder apprehension (*P* = 0.52 and 0.48 respectively). There was no difference in proprioception between operative and non-operative sides for the OL group (*P* = 0.43). Proprioception was poorer on the operative side for the OLCR group (*P* = 0.04) but better on the operative side for the AL group (*P* = 0.08). WOSI scores for the open surgical groups were similar (OL = 78, OLCR = 80, *P* = 0.91) and when combined (median WOSI = 79) demonstrated greater stability than the AL group (*P* = 0.009). There was no evidence of an effect of capsular repair or arthroscopic approach on the Walch-Duplay, Oxford Instability, or Rowe scores.

**Conclusions:**

There is no significant difference in ROM-ER 90 or WOSI score in patients who undergo the Latarjet procedure with and without capsular repair. The arthroscopic Latarjet may preserve proprioception but did not improve shoulder stability compared to the open Latarjet.

**Level of evidence:**

III, retrospective cohort study.

## Introduction

The shoulder is the most commonly dislocated joint in the body with 95% of shoulder dislocations occurring in the anteroinferior direction [1]. The “Latarjet” operation, originally described in 1954, is a surgical procedure used to manage bone loss from the anterior glenoid. It involves transferring the coracoid process with an intact conjoint tendon to the anterior neck of the glenoid [2]. Long term studies have shown it to be a successful surgical treatment for preventing re-dislocation in primary and recurrent anterior inferior shoulder instability [1–4]. This procedure has traditionally been performed through an open incision using the deltopectoral interval. In 2007 a technique was described to perform this procedure arthroscopically [1]. Whether performed through an open incision or arthroscopically, the Latarjet procedure has been shown to provide stability to the shoulder through two mechanisms: the sling effect of the conjoint tendon and the bone block [5–8]. Another possible stabilising mechanism is through augmentation with capsular repair either directly to the coracoacromial ligament or with bone anchors to the glenoid [9, 10]. Based on current literature, it is unclear whether capsular repair provides an added benefit. It is also unknown whether capsular repair may lead to restricted range of external rotation. The current literature examining these two questions is largely based on cadaveric studies with mixed results [11, 12, 8, 13].

The aim of this study is to evaluate the effect of capsular repair in the open Latarjet procedure on rotational range of active external rotation in 90 degrees abduction in the scapular plane and on post-operative apprehension, instability, proprioception and shoulder function scores. We also assessed the effect on these measures of open or arthroscopic procedures in patients who have not undergone capsular repair. Three groups were assessed, open Latarjet with no capsular repair (OL), open Latarjet with capsular repair (OLCR), and arthroscopic Latarjet without capsular repair (AL). Both the open and arthroscopic Latarjet procedures result in significant improvements in patient function and outcome scores, with low rates of recurrent instability and similar complication rates [14]. However, there has been no comparison of the outcomes of arthroscopic Latarjet with open Latarjet considering capsular repairs.

Our null hypothesis was that there would be no difference in post-operative range of motion, shoulder function scores, apprehension, or shoulder proprioception post operatively.

## Materials and methods

### Patients

We performed a retrospective multi-centre, multi-national cohort study on recruited patients with a minimum of 6-months follow-up post Latarjet procedure completed between 2016 and 2020. Ethics approval was sought and provided, and appropriate consent obtained from all patients. Data was collected from three participating orthopaedic surgical units in Australia and France from patients who had previously undergone a Latarjet procedure. The data collection process was undertaken from August 2019 through to October 2021. Data collection was conducted by the primary investigator. Patients were included if they had unilateral shoulder instability undergoing a Latarjet procedure between 2016 and 2020. Patients were excluded if they had less than 6 months post-operative follow up, an associated injury including a subscapularis tear, humeral avulsion of glenohumeral ligament (HAGL) or were unable to provide informed consent or adhere with the study protocol.

### Latarjet procedure

Patients who underwent an open Latarjet procedure, underwent a standard deltopectoral approach and a capsular split for glenoid exposure in line with the subscapularis fibres. The technique for Latarjet and repair of the capsule was based on the treating surgeon’s preference. Capsular repair was performed by either reattaching the capsule and labrum to the anterior glenoid margin using a bone anchor with sutures inside the bone block or suturing the coracoacromial ligament to the anterior capsular tissue on closure. The arthroscopic Latarjet procedure was performed as has previously been described [15]. A guide was used for graft and screw placement in the arthroscopic group and open group with capsular repair. The graft was placed freehand without a guide in the open group with no capsular repair. Two 3.5 mm partially threaded screws were used for fixation in all three groups.

### Outcome measures

The primary outcome, range of motion, was measured to examine external rotation at 90 degrees of shoulder abduction (RoM-ER90) using a Proteck goniometer (Proteck, Bern, Switzerland). The sample size of the study was based on the primary outcome of active external rotation in 90 degrees abduction. The mean predicted active ER in 90 degrees abduction 66 degrees with SD of 6.5. This is based on outcomes in the literature derived from Emami et al. (2011) and Rossi (2016) with a combined 130 patients analysed [16, 17]. Using G*Power 3.1.9.2 we needed at least 21 patients for a three-group analysis.

The secondary outcomes included were proprioception, apprehension and radiological outcomes and functional scores. Functional scores were assessed using the Western Ontario Shoulder Instability Index (WOSI) and Oxford shoulder, Oxford instability, Walch-Duplay and Rowe scores. Participants had objective clinical outcomes measured by the primary investigator. Clinical evaluation of shoulder apprehension was conducted using the apprehension/relocation test [18]. Assessment of shoulder proprioception was assessed by the method detailed by Glendon et al. [19]. This technique involves a laser pointer attached to the patient’s index finger standing 1 m away from a target on a wall. The patient actively moved to the target position (90° glenohumeral flexion), held for 5 s, returned their arm to their side and actively returned to the target position. A mean was calculated from three trials to provide an active relocation test (ART) score.

Post-operative CT scan analysis and radiographic review was conducted on participants and data was included if the scans had or could be conducted at greater than 3 months post-surgery. Data was recorded on bony union (greater than 50% bridging callus) and alpha angle. The alpha angle is measured between the screw axis and the glenoid rim and appropriate when ≤ 25°.

### Statistical analyses

The three groups, OL, OLCR, and AL, were compared descriptively with respect to age (median and interquartile range (IQR)), gender (frequency and % male), and handedness (frequency and % right-handed). Any general difference in age between groups was tested by a regression analysis and in proportion male or right-handed by Fisher’s exact test. Similarly, Fisher’s exact test was used to determine if the shoulder operated on varied across groups.

The effect of capsular repair was assessed by comparing the OL group with the OLCR group for each outcome measure. Normality of continuous outcomes was assessed by visual inspection and the Shapiro–Wilk test. As each variable was assessed to be non-normal, the Wilcoxon rank-sum test was used for the comparison. Union and apprehension were assessed using Fisher’s exact test. The effect of an arthroscopic approach was assessed by comparing the OL group with the AL group, similarly, using Wilcoxon rank-sum tests and Fisher’s exact tests.

For RoM-ER90 and proprioception, surgical and non-surgical sides were compared using the Wilcoxon sign rank test.

## Results

### Demographics

Fifty patients were recruited consecutively during the period of the study at all sites each undergoing a single Latarjet procedure. Six patients were excluded due to exclusion criteria as outlined above. Four had less than 6 months post-operative follow up, one patient had an associated injury, and one patient a humeral avulsion of glenohumeral ligament (HAGL). In total 44 patients across 3 sites were included for the final analysis. The mean follow up was 20 months (range 6 months to 3 years). Of the 44 patients, 31 were recruited from two sites in Australia operated on by two surgeons, and 13 from one surgical site in France operated on by one surgeon. The median age was 29.5 years with 91% being male across all three groups: 10 (90.9%) OL, 13 (100%) AL, and 17 (85.0%) OLCR (*P* = 0.35). Most patients were right-handed with no difference between groups; 9 (82.0%) OL, 13 (100.0%) AL and, 18 (90%) OLCR (*p* = 0.26) (Table 1). Overall, 55% of surgeries were performed on the right shoulder and this did not vary across groups; OL, 55%; OLCR, 55%, AL 54% (*P* = 1.00).

**Table 1:** Demographics of three groups

**Latarjet Surgery**	**Number**	**Age**^1^** (years)**	**Gender (Male %)**	**Handedness (Right %)**
Open, no capsular repair	11	30 (23, 31)	10; 91%	9; 82%
Open with capsular repair	20	27.5 (22.5, 37.5)	17; 85%	18; 90%
Arthroscopic, no capsular repair	13	31 (22, 40)	13; 100%	13; 100%
**All**	44	29.5 (22.5, 36.5)	91%	91%
***P* value**		*P* = 0.92	*P* = 0.35	*P* = 0.26

^1^Median (IQR)

### Range of motion

**Fig. 1:**
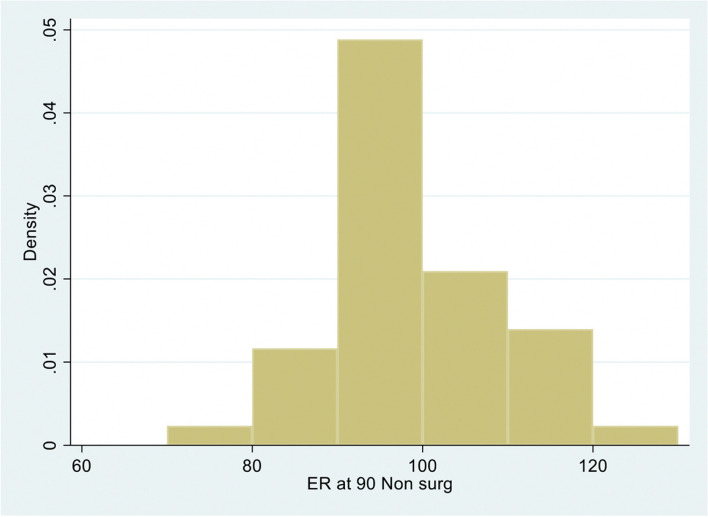
Distribution of range of motion of abduction in external rotation non-surgical side

**Fig. 2:**
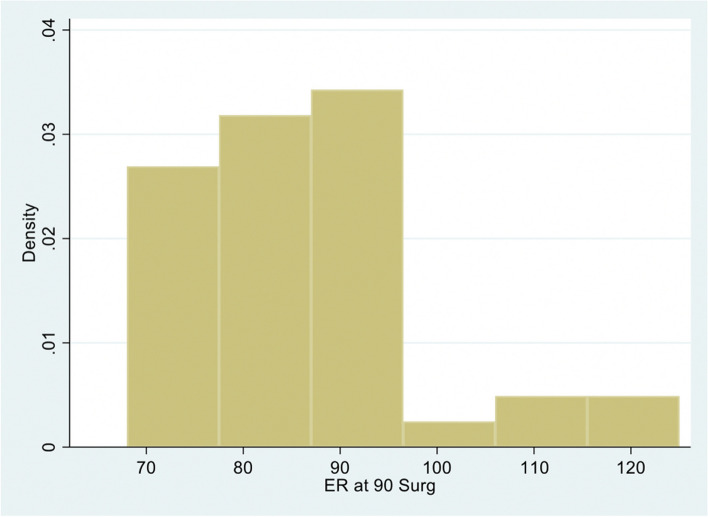
Distribution of range of motion of abduction in external rotation surgical side

The median (IQR) external range of motion at 90 degrees of abduction (RoM-ER90) on the non-surgical side (control) was 90 (90, 102), 95 (90, 100), and 94 (90, 100) degrees for OL, OLCR, and AL, respectively (Fig. 1, Table 2.) The median (IQR) for post-operative RoM-ER90 was greatest for the arthroscopic group at a median of 90 degrees (80, 95; *P* = 0.17 for arthroscopic approach). There was no appreciable effect of capsular repair in the open approach with a median of 78 (72, 90) for the OL group and 84 (75, 90; *P* = 0.87) for OLCR (Fig. 2, Table 2.). In each case, RoM-ER90 was less on the surgical side (OL *P* = 0.054; OLCR *P* = 0.0002; AL *P* = 0.085).

**Table 2:** Range of motion of external rotation in 90° abduction, Median (IQR); P_1,2_ – *P*-value for effect of capsular repair in open Latarjet; P_1,3_- *P*-Value for effect of arthroscopic surgery when there is no capsular repair

Range of movement (degrees) of external rotation in 90° abduction				
**Latarjet Surgery**	**Surgical side**	*P* value	**Non-surgical side**	*P* value
Open, no capsular repair (1)	78 (72, 90)		90 (90, 102)	
Open with capsular repair (2)	84 (75, 90)	P_1,2_ = 0.87	95 (90, 100)	P_1,2_ = 0.52
Arthroscopic, no capsular repair (3)	90 (80, 95)	P_1,3_ = 0.17	94 (90, 100)	P_1,3_ = 0.46

### Apprehension and proprioception

Neither capsular repair nor an arthroscopic approach appeared to affect the proportion of patients reporting shoulder apprehension (Table 3).

**Table 3:** Apprehension and proprioception; App. = Apprehension; Proprio. SS = Proprioception Surgical Side, Median (IQR); Proprio. NSS = Proprioception Non-Surgical Side’ Median (IQR). P_1,2_ – *P*-value for effect of capsular repair in open Latarjet. P_1,3_- *P*-Value for effect of arthroscopic surgery when there is no capsular repair

Apprehension and Proprioception						
**Latarjet Surgery**	**App**	*P* value	**Proprio. SS (cm)**	*P* value	**Proprio. NSS (cm)**	*P* value
Open, no capsular repair (1)	0/11		11.3 (9.0, 23.3)		11.1 (8.0, 20.5)	
Open with capsular repair (2)	2/19	P_1,2_ = 0.52	12.3 (10.0, 16.6)	P_1,2_ = 0.73	11.7 (7, 14)	P_1,2_ = 0.34
Arthroscopic, no capsular repair (3)	2/13	P_1,3_ = 0.48	10.8 (8.5, 12.7)	P_1,3_ = 0.36	12.6 (11.3, 16.2)	P_1,3_ = 0.81

**Fig. 3:**
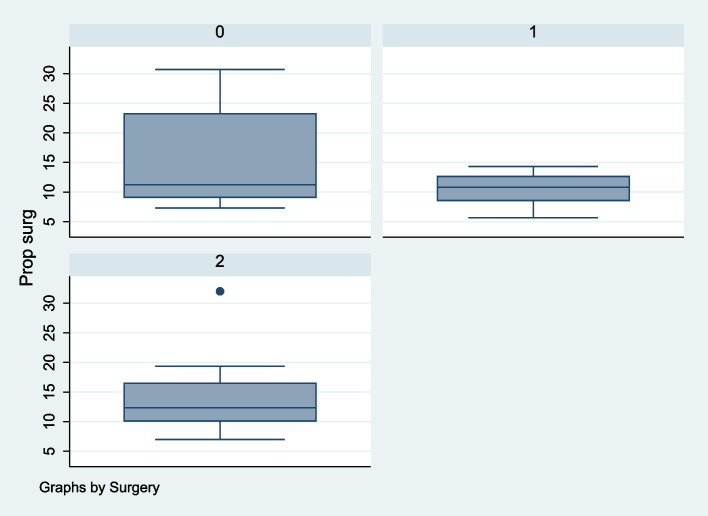
Interquartile range for proprioception post-surgical side. (0 = Open with no capsular repair, 2 = Arthroscopic, 3 = Open with capsular repair)

**Fig. 4:**
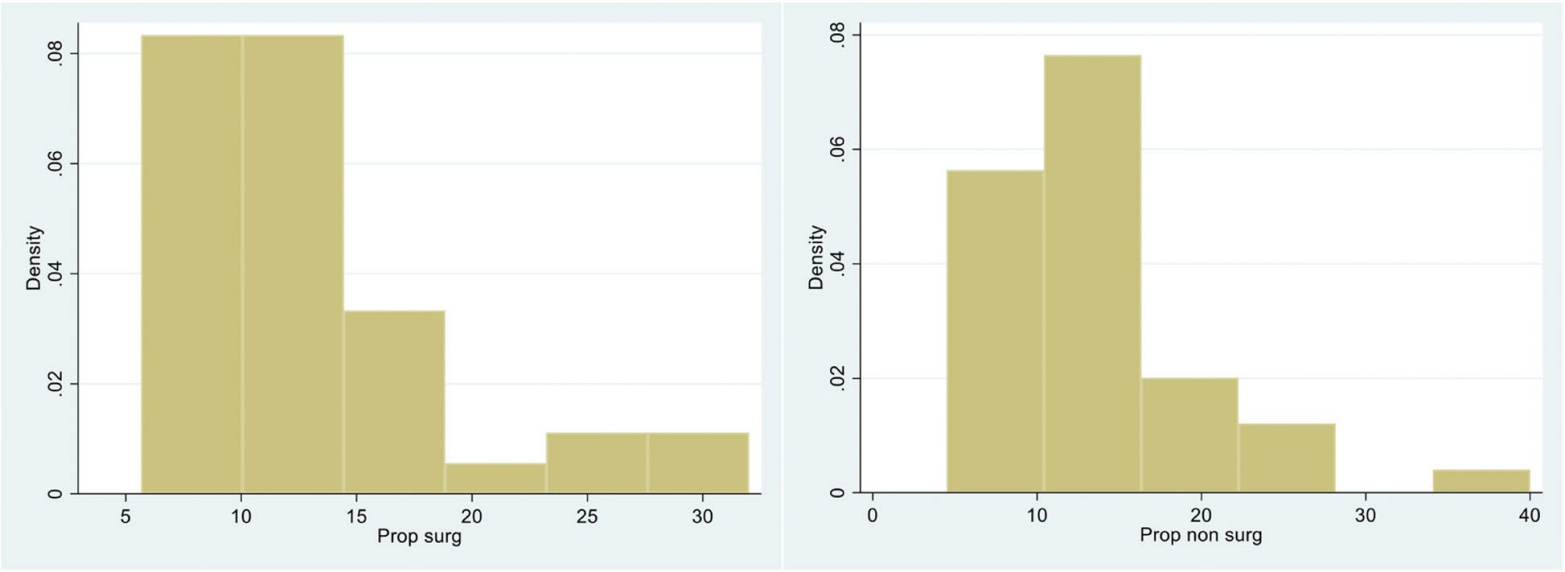
Distribution of proprioception surgical and non-surgical side

Proprioception was assessed in 10 OL, 12 AL, and 19 OLCR patients on the surgical side and 11, 12, and 19 on the non-surgical side respectively. Results are shown in Table 3. There was no evidence of a difference in proprioception between operative and non-operative sides for the OL group (*P* = 0.43) though proprioception was poorer on the operative side for the OLCR group (*P* = 0.04) but better on the operative side for the AL group (*P* = 0.08) (Figs. 3 and 4).

### Radiographic outcomes; bone union proportions and alpha angle

Out of a total of 44 patients, 28 had a CT scan or x-ray (anterior, posterior, lateral and axillary view) available for review at greater than three months post-surgery. All 28 patients (11/11 OL, 14/14 OLCR, 3/3 AL) had signs of union on post-operative imaging as evidenced by > 50% bridging callus across the graft site. Noting, however, that only 3 of 13 arthroscopic patients had union assessed. A total of 39 patients had a post-operative CT scan or axillary view x-ray where alpha angle could be measured: 11 OL, 20 OLCR, 8 AL. The computed tomography (CT) scan analysis of alpha angles is shown in Table 4.

**Table 4:** Bone union proportion; Union/Total, and alpha angle post-surgery. Median (IQR). P_1,2_ – *P*-value for effect of capsular repair in open latarjet. P_1,3_- *P*-Value for effect of arthroscopic surgery when there is no capsular repair

Radiographic Outcomes				
**Latarjet Surgery**	**Union**	***P* value**	**Alpha angle (degrees)**	***P* value**
Open, no capsular repair (1)	11/11		18.8 (12.5, 31.9)	
Open with capsular repair (2)	17/17	P_1,2_ = 1.00	8.7 (5.7, 13.3)	P_1,2_ = 0.003
Arthroscopic, no capsular repair (3)	3/3	P_1,3_ = 1.00	17.4 (11.4, 22.3)	P_1,3_ = 0.66

The smallest median angle was seen to be in the to be in the OLCR group (8.7 degrees) compared to the OL group (18.8 degrees; *P* = 0.003). The alpha angle in the arthroscopic group was 17.4 degrees being similar to the OL group (*P* = 0.66).

### Patient reported functional outcomes

These results are shown in Table 5. Western Ontario Shoulder Instability Index (WOSI) scores were higher (greater instability) in the AL group (median = 278) compared to the OL group (78, *P* = 0.14). The open surgical groups were similar (OLCR = 80, *P* = 0.91) and when combined (median WOSI = 79) demonstrated greater stability than the AL group (*P* = 0.009). There was no evidence of an effect of capsular repair or arthroscopic approach in the Walch-Duplay, Oxford Instability, or Rowe scores (Table 5). No dislocations were reported postoperatively in any of the groups during the study period.

**Table 5:** Patient reported functional outcomes. Median (IQR). P_1,2_ – *P*-value for effect of capsular repair in open latarjet. P_1,3_- *P*-Value for effect of arthroscopic surgery when there is no capsular repair

Latarjet Surgery	Western Ontario Instability Score	Walch- Duplay	Oxford Instability	Rowe
Open, no capsular repair (1)	78 (40, 420)	95 (80, 100)	46 (41, 48)	90 (75, 100)
Open with capsular repair (2)	80 (47, 180) P_1,2_ = 0.91	90 (70, 100) P_1,2_ = 0.33	44 (40, 47) P_1,2_ = 0.63	95 (90, 95) P_1,2_ = 0.55
Arthroscopic, no capsular repair (3)	278 (221, 316) P_1,3_ = 0.14	100 (90, 100) P_1,3_ = 0.47	42 (40, 46) P_1,3_ = 0.43	85 (75, 100) P_1,3_ = 0.42

## Discussion

The key finding in our study is that there was no appreciable difference in ROM-ER 90 in the open group between patients who did and did not have a capsular repair.

The arthroscopic group had a slightly greater range of ROM-ER 90 compared to the open Latarjet groups, but this did not reach statistical significance. The finding of no difference between capsular and no capsular repair is in keeping with a recent publication showing no difference in range of motion between the open Latarjet with and without capsular repair [20].

This study examined capsular repair using the modified Walch technique whereby the capsule is repaired to the CA ligament. It is important to note however that in our study, two differing techniques of capsular repair were performed. The technique of capsular repair in our study in eight of the twenty cases was performed with the capsule being fixed with a single anchor inside the bone block while the remaining cases used the Walch modification of suturing the coracoacromial ligament to the outside of the capsule. Repairing the capsule inside the bone block is analogous to augmenting the Latarjet procedure with an open Bankart type repair. Whilst our study had insufficient power to determine a difference between the two differing capsular repair techniques, previous studies have shown both arthroscopic and open Bankart repairs can reduce post-operative external rotation [21, 22]. Further studies are needed to compare whether repairing the capsule inside the bone block leads to a restriction in range of motion. It is also noted that a systematic review comparing intra-articular vs extra-articular bone block grafts in the Latarjet procedure showed a trend towards higher long-term rates of arthritis with intra-articular grafts [23].

A loss of range of motion may reduce performance in sports such as volleyball and freestyle swimming where a high degree of external rotation in abduction is required for correct technique. On the contrary, in contact sports such as Australian Rules Football (AFL) and Rugby Union repairing the capsule creates an extra barrier between the graft and humeral head during contact and ensures the bone block remains extra articular. A systematic review comparing intra-articular vs extra-articular bone block grafts in the Latarjet procedure showed a trend towards higher long-term rates of arthritis with intra-articular grafts [23].

Previous authors have compared re-dislocation rates between capsular shift and capsular repair procedure augmentation of the modified Bristow-Latarjet procedure [9] however to our knowledge no clinical studies have compared capsular repair vs no capsular repair in terms of post-operative range of motion and instability in the open and arthroscopic Latarjet procedures. Our study showed that the Latarjet procedure done with either of the three methods described has a high success rate with none of the participants in this study reporting post-operative re-dislocations.

Regarding apprehension, repairing the capsule inside of the bone block may also lead to reduced apprehension. Our study showed the highest rates of apprehension in the arthroscopic no capsular repair group. Further studies, in larger cohorts of patients will be required to definitively evaluate the effect of capsular repair and arthroscopic approach on post-operative apprehension.

Proprioception is important for shoulder function and reducing instability [24]. Mechanoreceptors within joint structures and capsular tissue are subject to injury during sport and surgical procedures. Damage to mechanoreceptors can lead to joint instability and deficits in neuromuscular control [24, 25]. Poor proprioception may impact on post-operative outcomes however research is lacking that assesses joint proprioception following the Latarjet procedure. When surgical and non-surgical sides were compared within groups of our study, arthroscopic surgery seemed to have a positive effect on proprioception whereas capsular repair in the open group was associated with poorer proprioception. Arthroscopic surgery may therefore cause less trauma and damage to mechanoreceptors which would explain its protective effect.

Performing an open Latarjet without capsular repair was associated with the highest interquartile range for proprioception in our study however, overall, there was no statistically significant difference between patients that had undergone an open or arthroscopic procedure with or without capsular repair. We did note that the proprioceptive data was not evenly distributed on the non-surgical and surgical sides with a similar trend for both sides in all groups. This likely suggests that if a patient has poor proprioception on one side, they are likely to have poor proprioception on the contralateral side. Again, further research with larger scale comparative studies are needed to examine the outcome of proprioception following the Latarjet procedure.

Our study showed a high union rate following the Latarjet procedure with all patients in our study going on to union confirmed by CT or radiographs at greater than 3 months follow up. The alpha angle was higher in the open group without capsular repair and arthroscopic group compared to the OLCR group (*P* = 0.003). Furthermore, the open groups had the lowest median WOSI scores compared to the arthroscopic group. Both these findings are in keeping with previous studies. A systematic review of 9 studies examining 956 patients demonstrated the postoperative WOSI score in the open Latarjet surgery was better than that of arthroscopic surgery and the α angle was smaller than the arthroscopic surgery group [26]. Whilst capsular repair is performed after the placement of screws and does not affect alpha angles, the use of a parallel drill guide has been shown to ensure more accurate placement of the coracoid graft in the axial and sagittal planes. In our study, the open group without capsular repair had the coracoid graft drilled and placed freehand whereas the other two groups had the graft placed with the use of guide. Despite the use of a guide in the arthroscopic group, the alpha angle remained high. This may be explained by the increased difficulty in achieving a parallel drill angle through the medial (M) portal during graft placement with the arthroscopic Latarjet procedure.

This study has a number of limitations. It is a retrospective study. There was no uniform clinical assessment of instability or radiographic assessment of preoperative bone loss and therefore this was not included. The procedures were performed by multiple subspecialist shoulder surgeons that would naturally have minor variations in their preferences. However, the principles outlined in the methods section were adhered to. Although including all available patients at participating sites, it has a small sample size and short follow-up of 6-months. There was no standardised post-operative rehabilitation protocol, and this may have also affected outcomes. Rehabilitation may have an effect of shoulder range of motion as well as outcome measures in the post-operative period.

## Conclusion

Overall, all three treatments proved effective in reducing dislocation resulting in good functional outcomes. There is no significant difference in ROM-ER 90 or WOSI score in patients who undergo the Latarjet procedure with and without capsular repair. The range of post-operative abduction external rotation was slightly higher when the shoulder capsule was repaired. The arthroscopic Latarjet may preserve proprioception but did not improve shoulder stability compared to the open Latarjet. There was no significant difference in apprehension. The post-operative WOSI score was slightly better for the open groups compared to the arthroscopic group. Bony union was achieved in all patients. Larger scale research is needed to examine the effects of capsular repair and different techniques on proprioception, apprehension, functional outcomes including sporting performance following the Latarjet procedure.

## Data Availability

The datasets used and/or analysed during the current study are not publicly available to protect study participant privacy but are available from the corresponding author on reasonable request.
